# Psychological, physical, and cognitive factors that influence tactical performance during a military relevant virtual reality scenario

**DOI:** 10.1186/s41235-025-00687-6

**Published:** 2025-11-04

**Authors:** Jennifer N. Forse, Meaghan E. Beckner, Grace E. Giles, Tad T. Brunyé, Marianna D. Eddy, Julie A. Cantelon, Mathias Basner, Christopher Connaboy, Bradley C. Nindl

**Affiliations:** 1 Neuromuscular Research Laboratory/Warrior Human Performance Research Center, Department of Sports Medicine and Nutrition, School of Health and Rehabilitation Sciences, University of Pittsburgh, 3860 South Water Street, Pittsburgh, PA 15203 USA; 2 Military Nutrition Division, United States Army Research Institute of Environmental Medicine, Natick, MA USA; 3 Cognitive Science and Applications Branch, United States Army DEVCOM Soldier Center, Natick, MA USA; 4 Center for Applied Brain and Cognitive Sciences, Tufts University, Medford, MA USA; 5 Unit for Experimental Psychiatry, Division of Sleep and Chronobiology, Department of Psychiatry, Perelman School of Medicine, University of Pennsylvania, Philadelphia, PA USA; 6 Center for Lower Extremity Ambulatory Research, Rosalind Franklin University of Medicine and Science, North Chicago, IL USA

**Keywords:** Coping, Military personnel, Decision-making, Virtual reality

## Abstract

Soldiers are challenged with interpreting information in unpredictable contexts, while maintaining high levels of job-specific performance. Virtual reality (VR) provides a controlled, immersive environment to evaluate military-relevant tasks under stress. This study determined psychological, physical, and cognitive associations with military-relevant VR task performance. Twenty-five male active-duty soldiers completed baseline psychological and cognitive assessments and then returned twice to complete VR-based Recognition Memory (RMT), Spatial Orienting (SOT), and Decision-Making (DMT) tasks under conditions of stress (active threat of torso electric shock) or no stress (torso vibration only). Baseline measures were categorized into 13 domains and standardized via z-scores. Generalized estimating equations were run with experimental condition (shock vs. vibrate) as the within-subject variable. Variables associated with correct object identification during the RMT include coping skills (e.g., acceptance), physical fitness (e.g., 2-mile run time), social intelligence, and personality traits (e.g., conscientiousness). Other coping skills (e.g., denial) decreased the odds of correct identification. Variables associated with accurate orienting on the SOT include coping skills (e.g., restraint), neurocognitive function (e.g., working memory), and prior video game experience. Additional measures of neurocognitive function (e.g., spatial orientation) reduced the odds of correct orientation. Variables associated with distinguishing targets during the DMT include coping skills (e.g., acceptance) and neurocognitive function (e.g., spatial orientation). Other coping skills (e.g., disengagement coping styles) reduced these odds. Coping skills, specifically higher acceptance, are associated with performance on military-relevant VR tasks and should be examined further to better understand how military performance could benefit from interventions targeting modifiable characteristics.

## Introduction

Stress is considered to be the relationship between a person and their environment and occurs when the person does not have the resources to deal with the demand of the environment (Folkman & Lazarus, 1985). Military operational environments can be very demanding and are often characterized as volatile, uncertain, complex, and, ambiguous (VUCA) (Nindl et al., 2018). As a result, military personnel must be able to manage stress and maintain high levels of cognitive acuity in VUCA environments. Research hypothesizes that by 2040, the cognitive-physical balance of demands at the tactical level will be primarily cognitive, again highlighting the importance of examining military personnel’s cognitive performance in military settings (Billing et al., 2021). It can be difficult to replicate the stress response observed in real-world military scenarios or gain access to military personnel undergoing intense training, so in order to safely examine how soldiers would perform on operational tasks while under conditions of stress and uncertainty, a virtual reality (VR) task was created (Brunyé & Giles, 2023). Virtual reality tasks may provide our military service members with virtual “combat immersions” that can be recorded and repeated until personnel are equipped with the skills needed to persevere in those scenarios (Scales, 2016).

A number of traits and characteristics have been associated with performance in military-relevant tasks (e.g., marksmanship, navigation, etc.) (Brunyé et al., 2024) and VR scenarios meant to simulate the stress and uncertainty of military environments (Giles et al., 2024). In a large analysis of trait level predictors of military performance (i.e., movement, shooting, communication, and navigation), most frequently predictive variables were physical, social/emotional, health, cognitive, and lifestyle variables (Brunyé et al., 2024). No variable was consistently predictive of performance across all realms of military performance, but some variables such as Army Combat Fitness Test (ACFT) performance, N-back working memory accuracy, emotion regulation, and grit were predictive of multiple areas of military performance (Brunyé et al., 2024). Some of these characteristics are unlikely to change (e.g., trait-like personality features), though others may be modifiable (e.g., state-like resilience) and serve as targets for future interventions to enhance performance during high-stress situations. However, factors to target for future interventions remain unclear, as some characteristics such as resilience and fitness have been associated with performance in military-relevant tasks (Brunyé et al., 2024), but not with performance in VR scenarios meant to elicit stress responses similar to those seen in military environments (Giles et al., 2024).

Stress exposure initiates a range of physiological responses in the body, particularly through neuroendocrine mechanisms such as the sympathetic–adrenal–medullary (SAM) pathway and the hypothalamic–pituitary–adrenal (HPA) axis (Beckner et al., 2022; Giacomello et al., 2020; Nater et al., 2005). These pathways are crucial in mediating the body's reaction to stressful stimuli, such as increasing heart rate and blood flow to muscle systems, and can display considerable variability between individuals based on factors like previous experiences and psychological resilience (Chu et al., 2019; Ebner & Singewald, 2017; Ozbay et al., 2007). Salivary alpha-amylase (sAA) and cortisol are two neuroendocrine markers often measured in response to stress and provide a noninvasive way to examine the stress response, especially over multiple measurements (Giacomello et al., 2020). Current literature suggests that a heightened stress response, characterized by higher concentrations of salivary cortisol and sAA, have been tied to poorer performance under high stress situations. Higher salivary cortisol concentrations, which is typically associated with prolonged stress exposure, have been associated with worse performance on a military-relevant laboratory-based memory task (Giles et al., 2024), worse instructor-rated military performance during advanced survival training (Morgan et al., 2004), and less recovery of cortisol concentrations after a combat casualty simulation was associated with worse performance in the simulation (McGraw et al., 2013). Previous research has shown sharp increases in sAA concentration levels after simulated stress (Brunyé & Giles, 2023; Valentin et al., 2015) and in the previous combat casualty simulation, a higher sAA response was also associated with worse performance in the simulation.

The effects of stress on performance are nuanced and may vary between traits, characteristics, and physiological responses of different individuals (Johnsen et al., 2012; Sekel et al., 2023; Wyss et al., 2016). Therefore, it is necessary to understand various factors that play a role in military performance under stress to better support and train soldiers. The objective of this analysis was to expand on previous research to examine a reduced battery of physical, psychological, cognitive, and demographic factors thought to be associated with cognitive performance during a military-relevant VR scenario (Brunyé et al., 2024; Giles et al., 2024). Based on previous findings of associations of stress responsiveness and military performance, this analysis also sought to examine the influence of neuroendocrine responses on variables associated with VR performance. Based on previous research, we hypothesized that better physical fitness, more accurate and faster responses on cognitive tests, better social intelligence, higher levels of resilience, and positive personality traits would be associated with better performance across three military-relevant VR performance tasks that examine memory, spatial orienting, and decision-making.

## Methods

### Participants

Twenty-five male active-duty soldiers (23.8 ± 4.7 years old, 26.4 ± 3.8 kg/m^2^) completed the study. Eligible participants were able to sit, stand, and move freely without pain and safely perform physical tasks. Participants were excluded if on a medical profile, self-reported history of back or lower-extremity problems, orthopedic injuries that limited range of motion, or history of cardiopulmonary or neurological pathologies. A detailed overview of subject demographics can be found in Table 1.

**Table 1:** Participants (*N* = 25) baseline characteristics across the 13 domains assessed for associations with virtual reality (VR) performance

Domain	Mean ± SD or *N* (%)
Demographics	
Age (years)	23.8 ± 4.7
Height (cm)	177.3 ± 7.5
Weight (kg)	83.2 ± 13.2
Body mass index (kg/m^2^)	26.4 ± 3.8
Video gamer	
Prior experience, yes/no (%)	13/12 (52%)
Military history	
Marksmanship experience, yes/no (%)	12/13 (48%)
Length of service (years)	2.9 ± 2.5
Number of deployments	0.3 ± 0.7
Weapons qualification Score	35.6 ± 4.7
Physical fitness	
ACFT score	363.5 ± 133.3
ACFT two-mile run time (min)	15.4 ± 2.9
Personality (NEO-FFI-3)	
Neuroticism	18.4 ± 5.7
Extraversion	30.7 ± 5.5
Openness to experience	28.8 ± 7.3
Agreeableness	28.7 ± 5.7
Conscientiousness	33.4 ± 4.7
Resilience	
CD-RISC score	76.1 ± 10.9
DRRI-II combat experiences score	18.6 ± 4.8
ER89 score	43.2 ± 5.8
PSS score	32.7 ± 4.9
Coping	
Positive reinterpretation and growth	11.5 ± 2.1
Mental disengagement	9.0 ± 2.3
Focus on and venting of emotions	7.5 ± 2.1
Active coping	11.0 ± 2.5
Denial	6.0 ± 2.6
Turning to religion	7.1 ± 3.7
Behavioral disengagement	5.5 ± 1.5
Restraint coping	9.6 ± 2.2
Seeking social support–emotional	7.7 ± 2.3
Substance use	5.5 ± 2.8
Acceptance	11.6 ± 2.6
Suppression of competing activities	9.5 ± 1.9
Matching-to-sample	
Correct responses	15.4 ± 3.5
Average correct response time (seconds)	5.4 ± 1.5
Average incorrect response time (seconds)	6.4 ± 2.1
Grammatical reasoning	
Correct responses	15.0 ± 6.0
Average correct response time (seconds)	3.9 ± 1.1
Average incorrect response time (seconds)	4.2 ± 0.8
Reading the mind in the eyes	
Lapses	1.4 ± 1.1
Correct responses	23.3 ± 3.2
Average response time (seconds)	7.7 ± 1.5
Cognition Reaction Time (RT)	
Motor praxis (MP) (ms)	770.3 ± 196.4
Visual object learning test (VOLT) (ms)	1993.4 ± 359.1
Fractal 2-back (F2B) (ms)	695.5 ± 111.3
Abstract matching (AM) (ms)	2444.9 ± 809.4
Line orientation test (LOT) (ms)	8932.6 ± 2329.2
Emotional recognition test (ERT) (ms)	3151.1 ± 902.1
Matrix reasoning test (MRT) (ms)	10,568.0 ± 3736.9
Digital-symbol substitution task (DSST) (ms)	1946.3 ± 261.8
Balloon analog risk test (BART) (ms)	1672.4 ± 1303.1
Psychomotor vigilance test (PVT) slowness (10 – 1/RT)	7.0 ± 0.2
Cognition accuracy	
Motor praxis (MP) (%)	29.8 ± 9.8
Visual object learning test (VOLT) (%)	85.8 ± 9.7
Fractal 2-back (F2B) (%)	69.0 ± 14.0
Abstract matching (AM) (%)	51.7 ± 11.8
Line orientation test (LOT) (%)	67.0 ± 9.7
Emotional recognition test (ERT) (%)	74.6 ± 15.5
Matrix reasoning test (MRT) (%)	61.7 ± 13.0
Digital-symbol substitution task (DSST) (%)	97.9 ± 3.4
Balloon analog risk test (BART) risk propensity (%)	66.2 ± 13.0
Psychomotor vigilance test (PVT) (%)	66.6 ± 13.4
Psychomotor	
Near-far quickness (NFQ) score	20.4 ± 7.4
Near-far quickness (NFQ) RT to near (ms)	1386.0 ± 531.6
Near-far quickness (NFQ) RT to far (ms)	1064.8 ± 386.2
Perception span score	32.8 ± 14.2
Multiple object tracking (MOT) composite score	1359.2 ± 452.4
Multiple object tracking (MOT) threshold speed (ms)	440.5 ± 100.2
Reaction time (ms)	343.7 ± 35.4

*ACFT* Army Combat Fitness Test, *NEO-FFI-3* NEO Five-Factor Inventory-3, *CD-RISC* Connor Davidson Resilience Scale, *DRRI-II* Deployment Risk and Resilience Inventory-II, *ER89* Ego Resiliency Scale, *PSS* Perceived Stress Scale, *ms* milliseconds

### Decision-Making under Uncertainty and Stress (DeMUS) virtual reality task

**Fig. 1:**
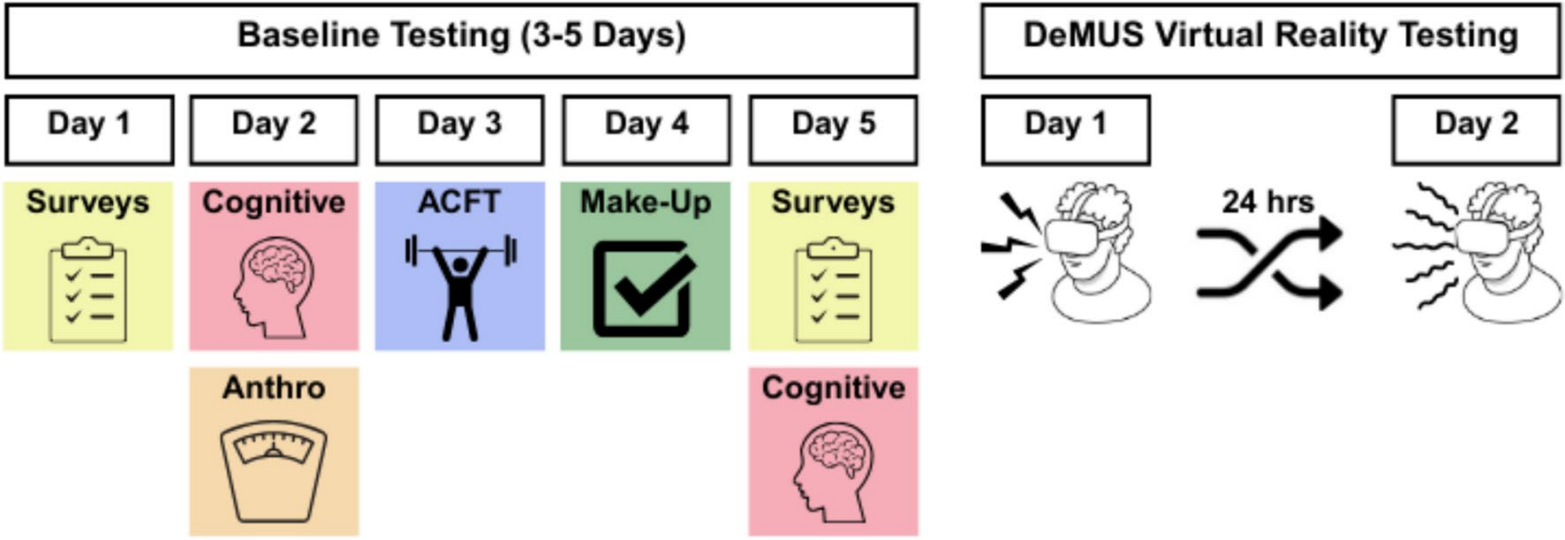
An example schematic of the study timeline including baseline testing and the Decision-Making under Uncertainty and Stress (DeMUS) virtual reality task. Baseline testing was completed over the course of 3–5 days before participants returned to the lab to complete the DeMUS task twice (once for each condition, shock vs. vibrate), separated by at least 24 h

Participants completed a series of baseline measures including psychological and cognitive assessments, described below, over the course of 3–5 days. Following baseline testing, participants returned to the laboratory for two additional visits, separated by at least 24 h, to complete the Decision-Making under Uncertainty and Stress (DeMUS) virtual reality task (Fig. 1), described in detail elsewhere (Brunyé & Giles, 2023). Both DeMUS testing sessions occurred in the morning, between 7:00 am and 11:00 am.

Briefly, the DeMUS VR task was created to elicit uncertainty and stress in a similar fashion to that experienced in military situations. The DeMUS task accomplishes this by stimulating physiological stress responses through a threat of shock (TOS) paradigm, inducing uncertainty by manipulating the clarity of stimuli introduced to participants during a decision-making task, and providing military-relevant performance metrics such as memory, spatial orientation, threat classification, and decision-making. The novelty of this scenario-based VR task is that it combines psychological, physiological, cognitive, and biochemical measures that can be used to examine a wide-ranging understanding of how stress and uncertainty impact performance on military style tasks. During each DeMUS session, participants wore a shock belt (StressX PRO Belt, StressVest, Winnipeg, Canada) around their torso. One session was set to a vibrate condition and one session set to a shock condition in a counterbalanced order between sessions and across participants. Participants initially completed a shock belt calibration to account for differences in pain tolerance of shock intensity levels. During calibration, participants placed the shock belt around their torso and were instructed to try each shock intensity level from the lowest setting (1), and to stop when they were no longer comfortable increasing the intensity. When the participant stopped and removed the belt, study staff recorded the highest intensity reached (e.g., 4) and one level below this (e.g., 3) was used for the DeMUS task.

Participants then completed Criterial Learning Tasks (CLT) as the first part of the DeMUS VR scenario to ensure that all participants have sufficiently and similarly learned all task-related stimuli for the three primary tasks in the DeMUS VR scenario (Brunyé & Giles, 2023). Following completion of the criterial learning task and 5 min of rest, the participant was fitted with the stress belt set to the appropriate shock condition (shock or vibrate) and intensity level determined during the calibration phase. Participants were then guided through the three immersive tasks within DeMUS.

*Criterial Learning Tasks (CLT)*. During these tasks, participants undergoing a learning phase of task-related stimuli that are needed to complete the three immersive VR tasks. During this learning phase, participants learn which targets they will need to locate in the Recognition Memory Task, a map of the virtual environment they will be placed in and need to be able to orient in during the Spatial Orientating Task, and how to distinguish different patterns of camouflage that delineate enemy from friendly targets in the Shoot/Don’t-Shoot Decision-Making Task.

**Fig. 2:**
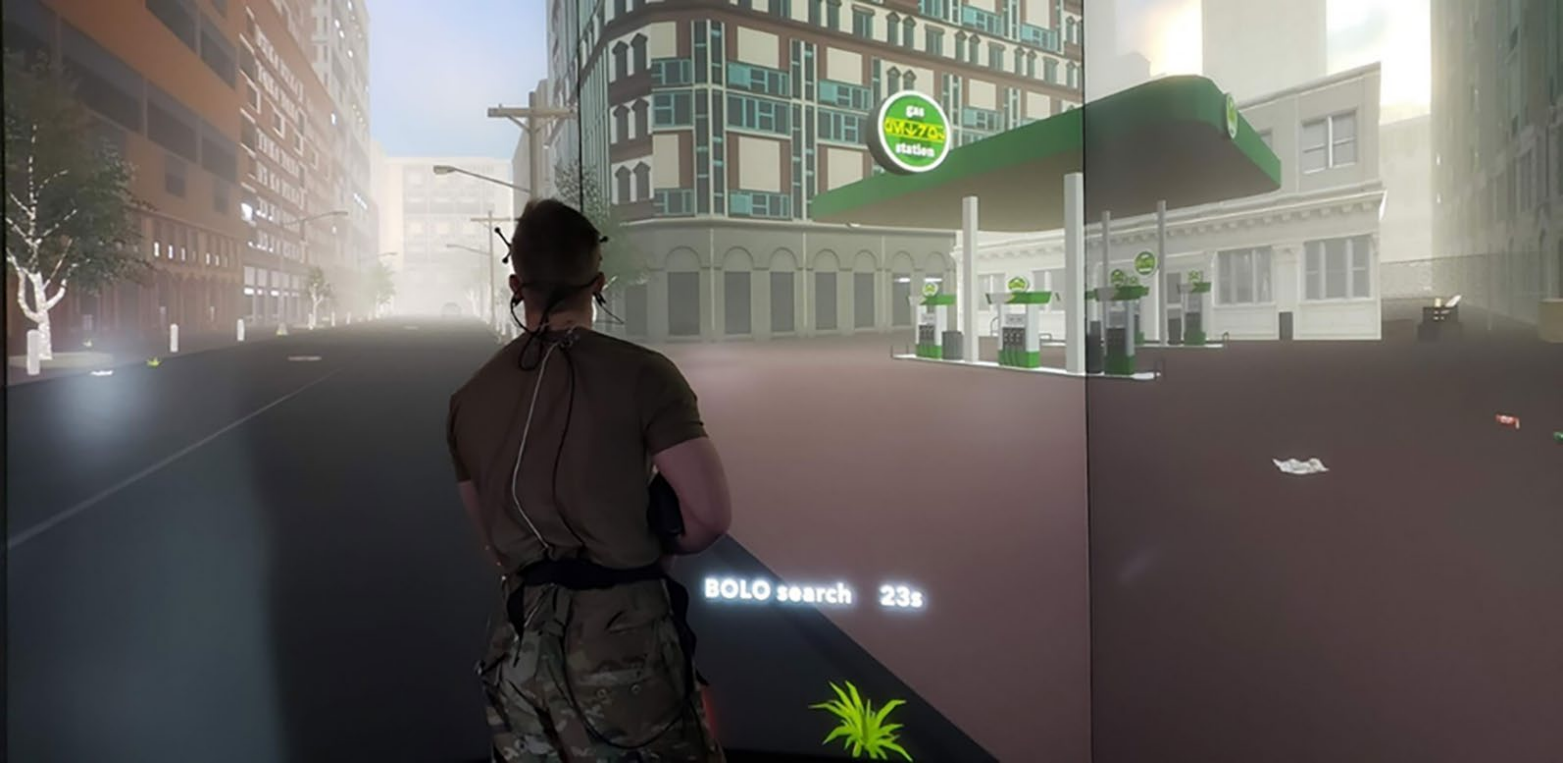
An example Recognition Memory Task trial, with the participant placed in front of the gas station in the virtual environment, patrolling in the environment for items on the Be On the Lookout (BOLO) list that they learned during the Criterial Learning Tasks (CLT) such as graffiti markings, vehicles, or bomb-making materials. Similar-looking items from the BOLO list that were not learned during the CLT are used as lures in this task

*Recognition Memory Task (RMT)*. During this task, participants are positioned in front of various landmarks within the virtual environment and tasked with locating targets within in the virtual environment they learned about beforehand in the CLT (e.g., people, vehicles, improvised explosive devices). In military terminology, this task requires identifying targets on a “Be On the Lookout” (BOLO) list (see Fig. 2). The RMT primarily demands episodic memory retrieval, requiring participants to distinguish previously studied (CLT-learned) targets from similar-looking lures. It relies on recognition-based decision-making (Cox & Shiffrin, 2017; Rugg & Yonelinas, 2003) and visual-perceptual discrimination (Magnussen & Greenlee, 1999; Scott et al., 2007), with possible working memory contributions in high-uncertainty conditions. For example, a learned image from the CLT that is on the BOLO list could be a dark blue truck with 8 wheels, while the lure is a lighter colored truck with only 6 wheels. The variable of interest from this task is discriminability, or DPrime, which is calculated as the z-score of hit rate minus z-score of false alarm rate. DPrime captures both sensitivity and specificity, and higher RMT DPrime scores indicate better performance on the task.

**Fig. 3:**
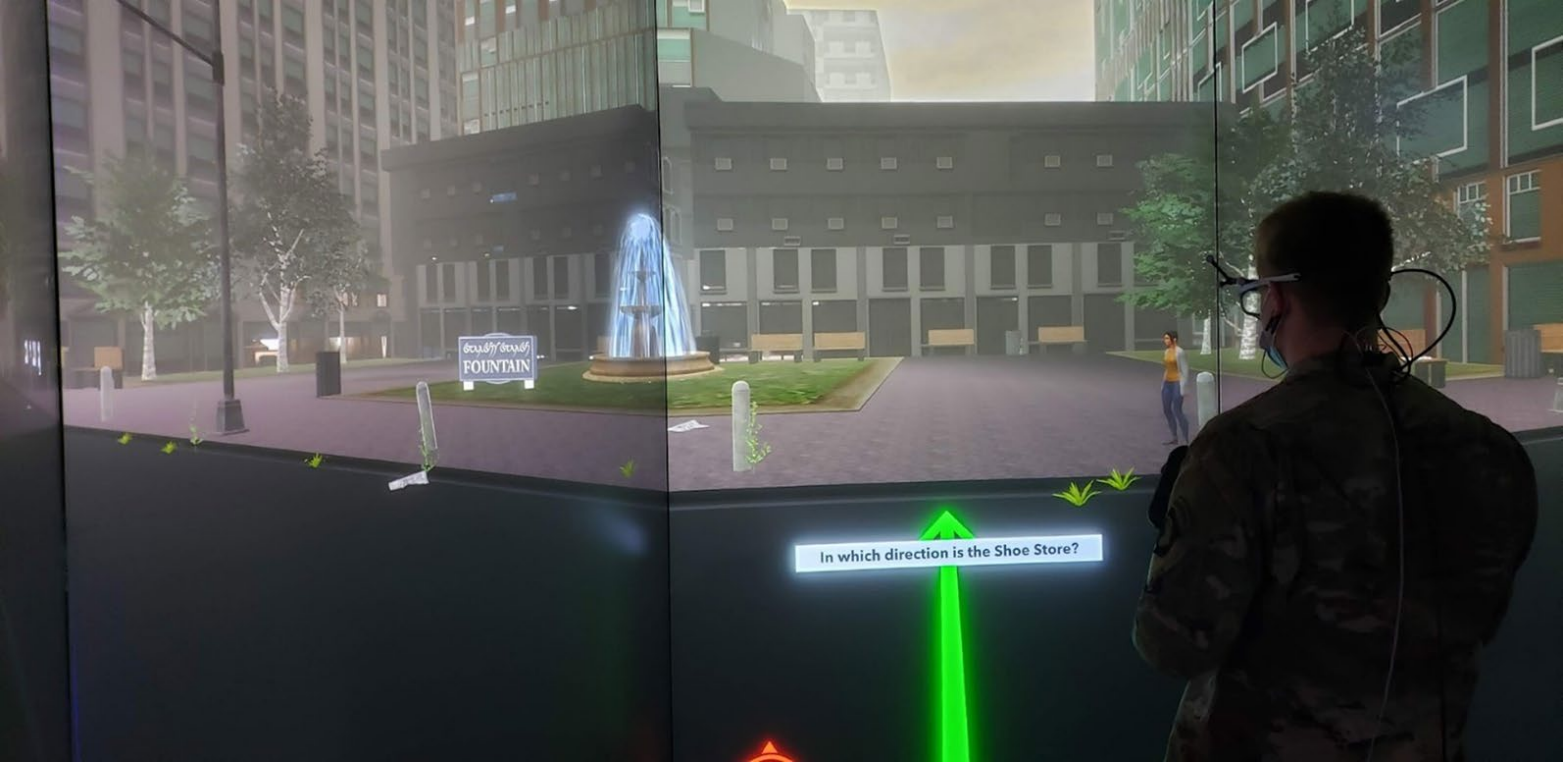
An example Spatial Orienting Task trial, with the participant placed in front of a landmark and instructed to position themselves toward another landmark using a joystick their gun. In this example, the individual was placed in front of the Fountain and instructed to position themselves toward the Shoe Store. A compass rose in red is placed at the bottom of the screen for the individual

*Spatial Orienting Task (SOT)*. During this task, participants are positioned in various places within the virtual environment (directly in front of the same landmark as the RMT) and instructed to orient themselves with a different location in the environment. For example, the participant may be placed next to the bowling alley and asked to point in the direction of the coffee shop (see Fig. 3). The SOT places demands on allocentric spatial memory, egocentric updating, and spatial perspective-taking. The task requires participants to mentally integrate previously learned maps with current egocentric viewpoints, orienting their body in large-scale virtual space, tapping into core processes of wayfinding and large-scale spatial orientation (Burgess, 2006; Wolbers & Hegarty, 2010). The variable of interest from this task is the point absolute direction error mean, which refers to the deviation of pointing direction vs. the actual direction of the landmark. Lower direction error indicates better performance on the task.

**Fig. 4:**
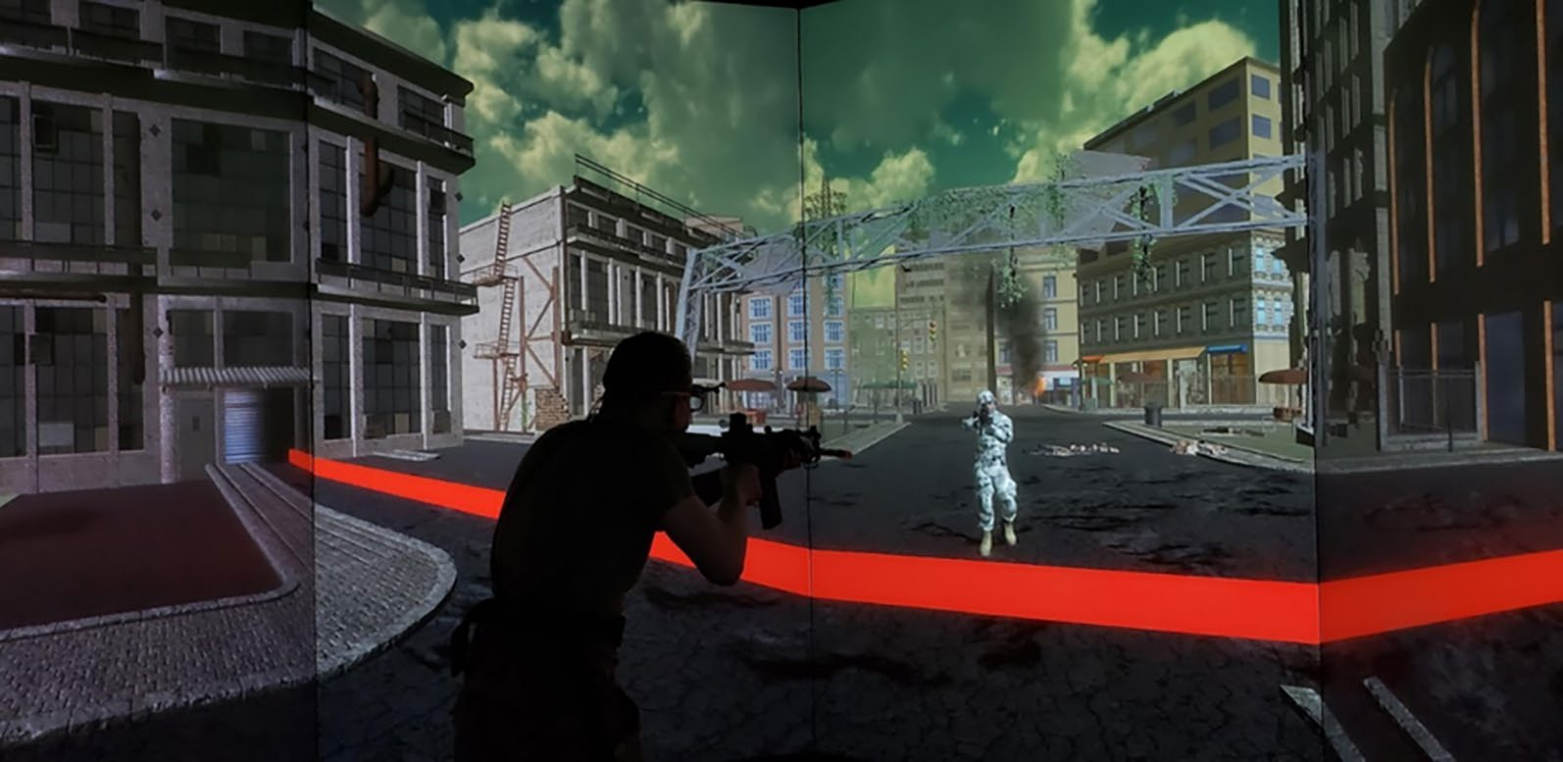
An example decision-making task trial, with a target in friendly or enemy camouflage approaching the participant. The participant has their gun raised in preparation of deciding whether to engage the target by pressing the trigger (if wearing enemy camouflage) or allow it to proceed past the red line (if wearing friendly camouflage)

*Shoot/Don’t-Shoot Decision-Making Task (DMT)*. During this task, participants are asked to distinguish friendly targets from enemy targets by examining the camouflage pattern of a virtual avatar (see Fig. 4). Participants learned the camouflage patterns during the CLT, and as an avatar styled as a soldier comes toward the participant in the environment, the participant must decide whether to engage the target (enemy) or allow it to proceed (friendly). The DMT requires perceptual decision-making under time pressure and uncertainty, drawing on pattern recognition, selective attention, and response inhibition to correctly identify threats based on camouflage (Brunyé & Gardony, 2017; Heekeren et al., 2004, 2008). This task may also tax working memory, as participants must retain and apply the rules learned during the CLT phase. The variable of interest in this task is also DPrime, calculated the same way as RMT DPrime. Similarly, higher DMT DPrime scores indicate better performance on the task.

### Ethical approvals and procedure

Healthy, active-duty soldiers were recruited from a larger cohort to participate in two studies, secondary analyses of which are reported herein: (1) the “Baseline” study characterizing Soldier cognitive, health, physical, and social–emotional traits (Giles et al., 2023) and (2) the DeMUS study characterizing cognitive performance under stress (Brunyé & Giles, 2023). A detailed overview of baseline participant characteristics can be found in Table 1.

The studies were approved by the Institutional Review Board at the U.S. Army Combat Capabilities Development Command Armaments Center (AC IRB #s 19-022 and 18-007) and Tufts University, as well the Army Human Research Protections Office. Written informed consent was obtained prior to any study activities.

### Psychological assessments

Participants completed the NEO Five-Factor Inventory-3 (NEO-FFI-3) to assess personality traits (Costa & McCrae, 1992), the Connor Davidson Resilience Scale (CD-RISC) to assess resilience (Connor & Davidson, 2003), the Combat Experiences scale of the Deployment Risk and Resiliency Inventory-2 (DRRI-2) to assess combat exposure (Vogt et al. 2013), the COPE Inventory (COPE) to assess coping strategies (Carver et al., 1989), the Ego Resiliency Scale (ER89) to assess contextual resilience (Block & Kremen, 1996), and the Perceived Stress Scale (PSS) to assess perception of stress over the past month (Cohen et al. 1983). A detailed description of each psychological assessment is provided in Supplementary Materials.

### Cognitive assessments

An adapted version of the Baddeley Grammatical Reasoning Test (GR) was used to assess language-based logical reasoning (Baddeley, 1968; Lieberman et al., 2016). The Matching-to-Sample test (MS) was used to assess working memory and pattern recognition (Lieberman et al., 2014; Shurtleff et al., 1994). The Reading the Mind in the Eyes (RMITE) test was used to examine social cognitive deficits and social intelligence (Baron-Cohen et al., 1997, 2001; Megías-Robles et al., 2020). The *Cognition* battery is comprised of 10 assessments and was used to examine: sensorimotor speed, spatial learning and memory, working memory, abstraction and concept formation, spatial orientation, emotional identification through facial expressions, abstract reasoning, complex scanning and visual tracking, risk decision-making, and vigilant attention (Basner et al., 2015; Moore et al., 2017). Four tests from the Senaptec Sensory Station^TM^ were used to assess psychomotor vigilance (Fraser et al., 2022). A detailed description of each cognitive assessment is provided in Supplementary Materials.

### Physical assessments

Physical assessments in this study were taken from the Army Combat Fitness Test (ACFT), and all events were completed as detailed in the US Army ACFT field testing manual (Department of the Army, 2018). The ACFT involves 6 events: a 3-repetition maximum deadlift, a standing power throw, hand release push-ups, a sprint-drag-carry, leg tucks, and a 2 mile run. The complete ACFT is estimated to take 54 min to complete, with approximately 34–37 min of active work.

### Physiological assessments

**Fig. 5:**
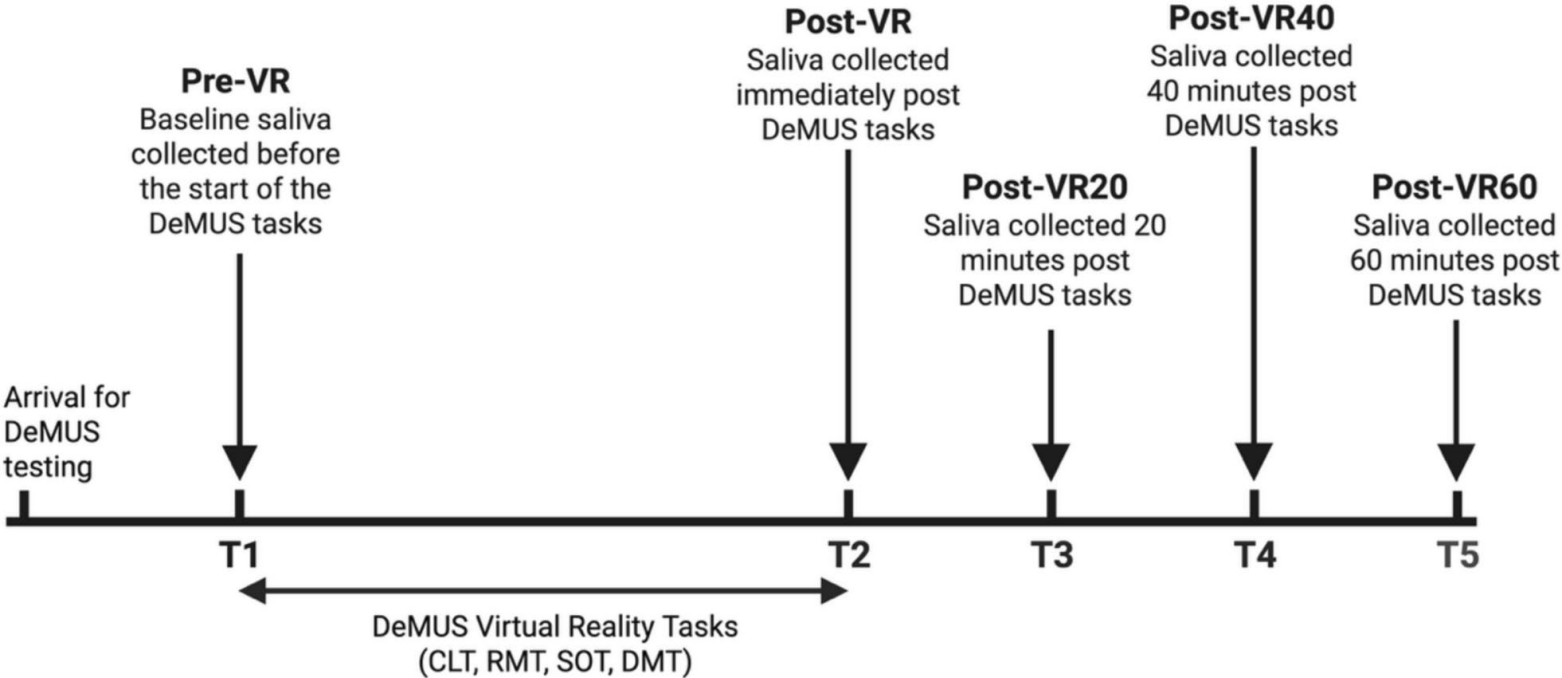
Timeline of biochemical specimen collection during the DeMUS testing sessions. Saliva was collected at 5 timepoints: T1) Pre-VR, T2) Post-VR, T3) Post-VR20, T4) Post-VR40, T5) Post-VR60

Salivary *α*-amylase (sAA) and cortisol were measured to assess SAM and HPA activation, respectively. Salivary samples were collected using the SalivaBio Oral Swab (Salimetrics, LLC, Carlsbad, CA) at five timepoints throughout completion of the DeMUS task: at rest prior to the DeMUS task (pre-VR), immediately following completion of the DeMUS task (post-VR), and 20 min (post-VR20), 40 min (post-VR40), and 60 min (post-VR60) following completion of the DeMUS task (Fig. 5). Participants were instructed to place the synthetic swab under their tongue for approximately 2 min to absorb adequate amounts of saliva. Enzyme-linked immunosorbent assays (ELISAs) were conducted for sAA and cortisol (Salimetrics, LLC, Carlsbad, CA). All samples were measured in triplicate with intra-assay coefficients of variation of 10% or less based on variance values reported by manufacturers. Sensitivity for each assay was: cortisol: < 0.007 μg/dL; sAA: 0.4 U/mL.

### Statistical analysis

VR performance outcomes were first analyzed using general linear mixed models with experimental condition (shock, vibrate) and condition order (vibrate then shock, or vice versa) included as fixed factors and participant included as a random intercept. Then, effect of biomarker response was analyzed using general linear mixed models with experimental condition as mentioned above, time (pre-VR, post-VR, -VR20, -VR40, and -VR60), and their interaction included as fixed factors and participant included as a random intercept. Condition order was also included as a fixed factor to identify potential carryover effects. Least significant difference post hoc testing was used to determine between- and within-group difference when significant main effects or interactions were identified. Residuals were examined to verify assumptions of normality and homogeneity of variance were met. Concentrations of cortisol and sAA did not meet these assumptions and were log-transformed prior to statistical analysis. Significance was set at *p* < 0.05.

Independent variables collected at baseline were categorized into 13 domains (Table 1), standardized using z-score conversions, and then assessed for correlations using Spearman correlation. Variables within the same domain that were strongly correlated (rho ≥ 0.7) were removed from further analyses. The relationship between standardized independent variables and performance on each DeMUS task was analyzed using individual generalized estimating equations (GEE) for each of the 13 domains, with participant included as a repeated measure and experimental condition included as the within-subject variable (Model 1).

Individual GEEs for each of the 13 domains (Model 1) were then re-analyzed with the addition of cortisol response to determine significant independent variables that may be mediated by cortisol (Model 2). Cortisol response was calculated as the relative change in cortisol from pre-DeMUS to 20 min post-DeMUS [(Post-VR20 – Pre-VR)/Pre-VR]. Independent variables that were significant in the original GEE (Model 1) but no longer significant in the cortisol GEE (Model 2) were assessed for correlations with cortisol response to determine the confounding (rho ≥ 0.7) or mediating effect of the independent variable.

Lastly, individual GEEs for each of the 13 domains (Model 1) were re-analyzed with the addition of sAA response to determine significant independent variables that may be mediated by sAA (Model 3). sAA response was calculated as the relative change in sAA from pre-DeMUS to immediately post-DeMUS [(Post-VR – Pre-VR)/Pre-VR]. Independent variables significant in the original GEE (Model 1), but no longer significant in the GEE including sAA response (Model 3) were assessed for correlations with sAA, to determine the confounding (rho ≥ 0.7) or mediating effect.

The purpose of adding cortisol and sAA to the domain-specific GEE models (Models 2 and 3) was not to establish a mediation pathway (from condition to biomarker to performance), but rather to explore whether biomarker reactivity altered the relationship between baseline traits and VR task performance. Time was included in these models to account for expected recovery-related variability in biomarker levels over the post-task timepoints and to reduce confounding in interpretation of biomarker effects. Because biomarker and condition may share variance, we interpreted only those cases where trait–performance relationships changed with biomarker inclusion but did not correlate strongly with biomarker response, suggesting a potential mediating (but not confounded) effect.

For all GEEs, significance was assessed using the false discovery rate (FDR) set at 10% to control for multiple comparisons and the Benjamini–Hochberg correction was applied to adjust the *p* values. Independent variables were considered statistically significant if their original *p* values were less than the adjusted threshold, maintaining an FDR of 10%. All analyses were conducted using SPSS (version 28; IBM SPSS Inc., Chicago, IL).

## Results

### DeMUS Task Performance

**Fig. 6:**
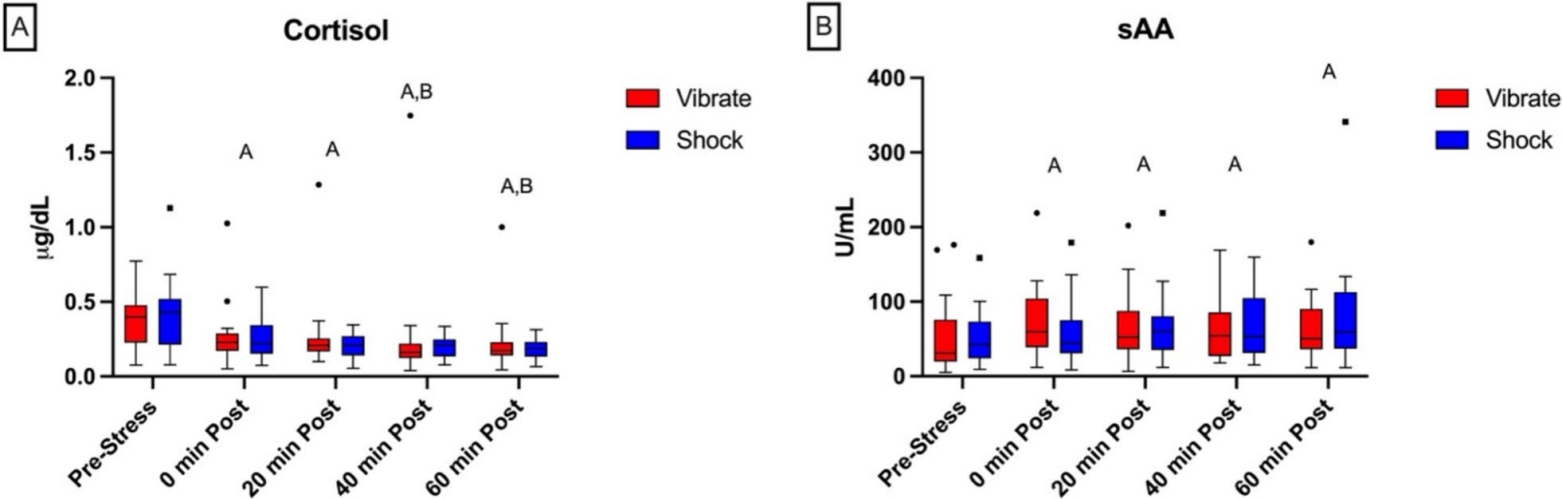
Stress responsiveness of salivary cortisol (**A**) and a-Amylase (**B**). Boxes indicate raw data median and interquartile range. Whiskers extend to 1.5 times the interquartile range, or to the minimum and maximum if no outliers are present. Outliers are presented as individual data points. Uppercase letters (A,B) represent least significant difference pairwise comparisons for the main effect of time. *A* = significantly (*p* < 0.05) different than Pre-Stress. *B* = significantly different than 0 min Post

Performance during the DeMUS task did not differ between shock or vibrate conditions for RMT (*p* = 0.144), DMT (*p* = 0.630), or SOT (*p* = 0.280). Cortisol concentrations were lower at all timepoints after the DeMUS task compared to before the task (all *p* < 0.001) and did not differ between shock and vibrate conditions (*p* = 0.920; Fig. 6A). sAA was elevated at all timepoints after the DeMUS task compared to before the task (all *p* < 0.001) with no difference between shock or vibrate conditions (*p* = 0.337; Fig. 6B). No significant condition order or interaction effects were observed. Thus, our stress induction did not appear to alter HPA axis activity (as assessed via salivary cortisol), and the DeMUS task generally appeared to increase SAM activity (as assessed via sAA) in both the stress and vibrate conditions.

### Significant associations with Recognition Memory Task performance

A total of 16 baseline variables were strongly correlated (rho ≥ 0.7) with other variables within the same baseline domain and removed from subsequent analyses (Supplemental Table 1). Ten variables were significantly associated with RMT performance: 5 measures of coping, 2 measures of physical performance, 2 measures of social intelligence, and 1 measure of personality (Table 2). Higher scores of COPE Acceptance and Seeking social support—emotional, greater physical test scores and 2-mile run times, better accuracy and more lapses in social intelligence (RMITE), and higher scores of conscientiousness increased the odds of successfully identifying the target in the virtual environment. Higher scores of COPE Suppression of competing activities, Focus on and venting of emotions, and Denial decreased the odds of successfully identifying the target. In the coping domain of Model 1, DeMUS condition was a significant factor, with shock associated with poorer performance on the RMT (*β* = − 0.74, 95% CI [0.252, 0.903], *p* = 0.023). In other words, when controlling for all coping variables, DeMUS condition significantly altered RMT performance. When relative change in cortisol (Model 2) was assessed in the coping domain, Acceptance coping style was no longer a significant predictor of RMT performance, suggesting that cortisol responses mediate the relationship between Acceptance coping style and RMT performance. No other changes were observed with the inclusion of cortisol or sAA in the models. Spearman correlations between key coping variables, cortisol and sAA reactivity, and VR task performance outcomes can be found in Supplementary Table 2.

**Table 2:** Significant associations with RMT Performance

Predictor	Wald statistic	*B*	95% CI	*p* value
COPE suppression of competing activities	20.634	− 1.282	− 1.835, − 0.729	< 0.001
COPE focus on & venting of emotions	9.691	− 0.971	− 1.582, − 0.359	0.002
COPE denial	7.463	− 0.766	− 1.315, − 0.216	0.006
ACFT score	5.154	0.491	0.067, 0.915	0.023
COPE acceptance	5.002	0.509	0.063, 0.955	0.025
ACFT two-mile run time	9.281	0.612	0.218, 1.005	0.002
RMITE lapses	4.692	0.619	0.059, 1.180	0.030
RMITE number correct	5.421	0.678	0.107, 1.249	0.020
NEO conscientiousness	7.473	0.833	0.236, 1.431	0.006
COPE seeking social support–emotional	10.619	0.914	0.364, 1.464	0.001

*ACFT* Army Combat Fitness Test, *RMITE* Reading the mind in the eyes, *CI* confidence interval

### Significant associations with Spatial Orienting Task performance

Eleven variables were significantly associated with SOT performance: 6 measures of neurocognitive function, 3 measures of coping, 1 measure of logical reasoning, and prior video game experience (Table 3). Higher scores of COPE Acceptance, Restraint and Behavioral disengagement, greater accuracy in working memory (F2B), psychomotor vigilance (PVT) and language-based logical reasoning (GR), faster emotion recognition (ERT) response time, and prior video game experience were all associated with reduced angular error (i.e., improved performance) when making orientation estimates. Greater accuracy of complex-scanning and visual tracking (DSST) and slower response times in spatial orientation (LOT) and sensorimotor speed (MP) assessments were associated with increased angular error when making orientation estimates. DeMUS condition (i.e., shock versus vibrate) was non-significant across all domains in all three models for SOT performance. All significant variables remained significant with the inclusion of cortisol or sAA in the models.

**Table 3:** Significant associations with SOT Performance

Predictor	Wald statistic	B	95% CI	*p* value
COPE acceptance	16.535	− 16.301	− 24.158, − 8.444	< 0.001
COPE restraint coping	15.264	− 13.649	− 20.496, − 6.802	< 0.001
COPE behavioral disengagement	12.890	− 12.136	− 18.761, − 5.511	< 0.001
F2B accuracy	14.346	− 11.950	− 18.133, − 5.766	< 0.001
GRAM correct responses	10.350	− 11.082	− 17.834, − 4.331	0.001
Video gamer	9.003	− 8.236	− 13.616, − 2.856	0.003
ERT RT	6.926	− 7.836	− 13.673, − 2.000	0.008
PVT accuracy	10.934	− 6.597	− 10.508, − 2.687	0.001
DSST accuracy	7.985	6.872	2.106, 11.639	0.005
LOT RT	9.524	9.806	3.578, 16.034	0.002
MP RT	8.606	14.488	4.808, 24.168	0.003

*F2B* fractal 2-back (Cognition), *GRAM* grammatical reasoning, *ERT* = emotion recognition test (Cognition), *PVT* psychomotor vigilance test (Cognition), *DSST* digital symbol substitution task (Cognition), *LOT* line orientation test (Cognition), *MP* motor praxis (Cognition), *RT* reaction time, *CI* confidence interval

### Significant associations with Decision-Making Task performance

Eight variables were significantly associated with DMT performance: 7 measures of coping and 1 measure of neurocognitive function (Table 4). Higher scores of COPE Acceptance, Denial and Focus on and venting of emotions and slower response times in spatial cognition (LOT) were associated with increased odds of distinguishing friendly from enemy targets. Higher scores of COPE Seeking social support—emotional, Substance use, and Mental and Behavioral disengagement were associated with decreased odds of distinguishing friendly from enemy targets. LOT response time was no longer significantly associated with DMT performance when relative change in cortisol (Model 2) or sAA (Model 3) was included in the spatial orientation model, suggesting that physiological stress responses mediate the relationship between LOT and DMT performance. No other changes were observed with the inclusion of cortisol or sAA in the models.

**Table 4:** Significant associations with DMT Performance

Predictor	Wald statistic	B	95% CI	*p* value
COPE seeking social support–emotional	36.260	− 1.592	− 2.110, − 1.074	< 0.001
COPE mental disengagement	13.471	− 1.085	− 1.665, − 0.506	< 0.001
COPE behavioral disengagement	14.423	− 0.873	− 1.324, − 0.423	< 0.001
COPE substance use	9.980	− 0.826	− 1.338, − 0.313	0.002
LOT RT	5.858	0.566	0.108, 1.024	0.016
COPE acceptance	30.500	1.288	0.831, 1.745	< 0.001
COPE denial	24.035	2.075	1.246, 2.905	< 0.001
COPE focus on & venting of emotions	41.539	2.084	1.450, 2.718	< 0.001

*LOT* line orientation test (Cognition), *RT* reaction time, *CI* confidence interval

## Discussion

The primary finding from this randomized, crossover study was that baseline psychological assessments, specifically coping skills, were the domains most associated with overall performance across all tasks of the DeMUS VR scenario. Of the 29 variables that were observed to be associated with performance, coping skills made up half of those variables across all three tasks, demonstrating that these modifiable coping skills may be an important factor in stressful situations and occupations and therefore could benefit from targeted interventions. Interestingly, some of the traits and characteristics were associated with performance on more than one of the three tasks, but the relationship differed between tasks. Because the three tasks examined three different facets of cognitive performance (i.e., memory recall in the RMT, spatial orientation in the SOT, and decision-making in the DMT), these results may provide insight on how future interventions may need to be task dependent rather than focused on an overall scenario.

Finally, in this study, stress biomarkers did not appear to play a significant role in VR performance, however, that may be due to previously reported low levels of HPA activation during the DeMUS task (Brunyé & Giles, 2023; Giles et al., 2024). No significant differences have been reported between the shock or vibrate conditions of the DeMUS task, which were the same results seen in this investigation (Brunyé & Giles, 2023). However, like previous reports, this investigation did show significant changes in stress biomarkers in response to the DeMUS task (Fig. 6), possibly suggesting that while the DeMUS task is not sensitive enough to elicit differences in the anticipatory stress of the shock vs. vibrate conditions, it does elicit an overall stress response. Taken together, these findings suggest that modifiable psychological characteristics such as coping skills are associated with performance on military tasks; however, given varying directions of relationships, any intention of future interventions may be best suited to target specific tasks, rather than overall military-relevant performance.

### Coping skills

Coping skills were the domain that was most associated with performance across all three VR task outcomes. Several coping skills were associated with performance on multiple VR tasks (i.e., Denial, Behavioral disengagement, Seeking social support–emotional), but Acceptance was associated with performance across all three tasks. Folkman and Lazarus (1980) identified two primary styles of coping, problem-focused and emotion-focused, while Carver et al. (1989) took it a step further to identify coping-styles that were useful and “less useful” strategies. Problem-focused coping styles (e.g., Suppression of competing activities and Restraint) deal with the source of the stressor while emotion-focused coping styles (e.g., Acceptance, Denial, and Seeking social support–emotional) deal with the thoughts and feelings that are associated with the stressor (Folkman & Lazarus, 1985). It is believed that the use of problem- or emotion-focused coping skills to deal with stress may be contextual in nature, and that one is not more useful than the other. For example, using a problem-focused coping skill like guided relaxation (i.e., active coping) to reduce stress may be useful prior to a fitness test while during the fitness test, it may be more beneficial for the individual to use an emotion-focused coping skill such as positive self-talk (i.e., positive reinforcement) to improve their performance (Sideridis, 2006). But there are some coping skills that are considered maladaptive and “less useful” (e.g., mental and behavioral disengagement, and focus on & venting of emotions), though research suggests that too much reliance on any one kind of coping skill may eventually lead to dysfunction (Carver et al., 1989). Indeed, it seems as though the use of both problem-focused and emotion-focused coping skills is the most important and has an effect on performance in academic settings and thus may extend to other performance settings (Sideridis, 2006). As a whole, emotion-focused coping skills had the highest number of associations and problem-focused coping skills had the lowest number of associations with performance across all three VR tasks, suggesting that participants in this study tend to deal with the thoughts and feelings of the stressor more than they deal with the source of the stressor. Interestingly, less useful coping skills had the second most associations with performance across all VR tasks. These results may mean that soldiers rely on emotion-focused and less useful coping skills when undergoing military operations, highlighting that more research is needed to understand to complex interplay between coping skills and performance on military operational tasks. These results provide preliminary evidence that there are relationships between coping skills and performance on a variety of military tasks, but a more detailed examination into the relationships between specific coping skills, different coping styles, and performance on even more military tasks is necessary.

### Task dependency

There are a number of variables, mostly coping skills, that were associated with performance on multiple tasks, however, in contrasting ways. These results may suggest a potential task-dependent relationship between specific traits or characteristics and a desired performance outcome, rather than overall performance. Unfortunately, research on the relationships between specific coping skills and cognitive functions is not well studied, and the current study did not examine how specific coping skills were used during the cognitive tasks.

A “less useful” coping skill, behavioral disengagement, was associated with better performance on VR spatial orientation (SOT) but worse performance on VR decision-making (DMT). Previous research suggests that the relationship between disengagement coping skills, such as Behavioral disengagement, and decision-making may be a more complex, feedforward-feedback process model that moves from a stressor (e.g., threat of shock), to a physiological response, to primary appraisal of the stressor and the consequences (e.g., poor decision-making), that lead to the secondary appraisal and use of coping skills (e.g., behavioral disengagement) and the reinforcement of this coping skill to cope with similar stressors (Korosec-Serfaty et al., 2022). Soldiers in this study may rely on disengagement coping skills and therefore score higher on the COPE Behavioral disengagement subscale, which in turn can lead to poor decision-making during the DMT.

The COPE subscales Focus on and venting of emotions and Denial were associated with better performance on VR decision-making (DMT), but worse performance on the VR memory recall (RMT). A previous study examining how a team of police officers utilized coping skills to minimize uncertainty in decision-making during a highly stressful event (i.e., hostage negotiation) found that the use of suppressive coping skills (e.g., Denial) was only used during the assessment of a situation, and led to a temporary omission of a decision, but not the decision-making aspects of the scenario (van den Heuvel et al., 2014). Therefore, the relationship that we observed between Denial coping and decision-making may be in the initial assessment of the friendly or enemy individual in the DMT trial, which has an effect on overall performance on the task.

In contrast to the previously mentioned task-dependent relationships, the COPE subscale Seeking social support–emotional was associated better performance during VR memory recall, but worse performance during VR decision-making. One suggestion for this result comes from research examining emotional support from parents and teachers on children’s working memory performance. Vandenbroucke et al. (2017) reported that parents and teachers who provided adequate amounts of emotional support had a significant influence on children’s working memory performance. While this finding is in a population that is much different than the Soldiers examined in the current study, results from this study may provide evidence that emotional support has an effect on working memory even after maturation.

There were no variables that showed task dependency between the RMT and SOT, suggesting that factors that are associated with better performance in the memory recall scenario do not negatively impact performance in spatial orientation scenario and vice versa. To further contextualize these task-dependent relationships, it is important to consider the distinct cognitive demands that each task imposes. The RMT engages recognition memory and visual discrimination, requiring retrieval of learned visual details under potentially distracting conditions. The SOT, in contrast, taxes large-scale spatial memory, mental rotation, and egocentric perspective-taking, cognitive processes more closely aligned with wayfinding and spatial updating. The DMT emphasizes rapid perceptual categorization and decision-making under uncertainty, likely involving selective attention, inhibition, and learned rule application. These distinct demand characteristics may help explain why particular coping strategies and cognitive abilities show differential associations across tasks, and they underscore the value of treating military-relevant cognitive performance as task-specific rather than uniform.

### Role of biomarkers in VR task performance

In this analysis, the DeMUS scenario elicited stress biomarker responses similar to those seen in previous research. For example, in this analysis, sAA increased over across all timepoints in response to the scenario, which follows a similar pattern as the responses seen in the development of the scenario (Brunyé & Giles, 2023). Salivary cortisol did not follow the same patterns, but this may be due to the methods of data collection. In the development of the scenario, participants attended each session at the same time of morning to account for diurnal variation; however, in this analysis data was collected at varied times, and therefore this pattern would be expected given diurnal reductions in cortisol before mid-day. While these results suggest a stress response was elicited by the DeMUS scenario by an increase of sAA, there was no evidence that the scenario invoked different responses to the two different stress conditions (shock vs. vibrate).

We note a conceptual complexity in interpreting the influence of cortisol and sAA responses, given that both were expected to vary as a function of the experimental condition (shock vs. vibrate). However, our analytic goal was not to establish a causal mediation pathway from experimental manipulation through physiological reactivity to task performance. Rather, we aimed to explore whether these physiological responses may account for or modify the relationships between baseline traits and performance. Including time in the models allowed us to disambiguate physiological reactivity from temporal patterns of biomarker return to baseline. Thus, while we acknowledge potential shared variance between condition and biomarkers, our approach prioritized detecting shifts in trait-performance associations that may suggest physiological mediation, rather than modeling complete pathways or causal chains.

In this analysis, neither the cortisol nor sAA response to the VR scenario was a significant factor in VR performance, but results may suggest there is a mediating relationship between certain traits or characteristics and performance. Specifically, COPE acceptance was no longer significantly associated with memory recall (RMT) performance when relative change in cortisol was included in the model and spatial orientation (LOT) reaction time was no longer associated with VR decision-making (DMT) when relative change in cortisol or sAA were included in the GEE model. These variables were not significantly correlated with the biomarker responses, suggesting that the biomarker responses were not confounding variables, but rather may be mediating the relationship between these baseline characteristics and VR performance.

It has been reported in previous literature that there is a relationship between stress reactivity and acceptance coping (O’Malley et al., 2024), and more specifically that the use of acceptance as a coping style was negatively associated with individuals' cortisol response throughout the day, highlighting acceptance as an adaptive coping response (Turner-Cobb et al., 2010). Cortisol concentrations have also been linked to recognition memory performance, specifically high stress reactivity has been linked to reduced performance on recognition memory tasks (McCullough et al., 2015; Shields et al., 2017). Previous research has not yet examined the relationships between coping skills, stress reactivity, and memory performance together, but the current results suggest that the effect of acceptance as a coping skill on recognition memory task performance may be, at least partially, due to changes in cortisol.

Spatial orientation performance, specifically response time, on a VR task was associated with SAM axis response to stress, but not HPA axis response, in a study examining the effect of an acute stressful task on spatial performance (Richardson & VanderKaay Tomasulo, 2011). It is important to note that in this study, SAM activation was measured using heart rate and blood pressure assessments instead of biomarkers of stress. Less research exists that specifically examines cortisol’s relationship with spatial orientation, though substantial literature exists that ties cortisol reactivity with other cognitive functions such as spatial learning and attention (Richardson & VanderKaay Tomasulo, 2022; Schwabe et al., 2009). However, the relationship between levels of cortisol and decision-making is more well known, with previous research pointing to a negative association between cortisol and performance on a military decision-making scenario and stress reactivity, measured by cortisol concentrations, being predictive of future decision-making competence (Duque et al., 2022; Shields et al., 2016; Winslow et al., 2015). The relationship between salivary alpha-amylase and decision-making has been less extensively studied; however, a recent study reported an association between sAA and impulsive decision-making during a scenario with uncertain outcomes, similar to the DMT of the DeMUS scenario (Takahashi et al., 2007). Again, while the relationships between spatial orientation and HPA/SAM responsiveness as well as stress responsiveness and decision-making have been examined in separate studies, the relationships between all three have yet to be examined. This study serves as first evidence of the potential mediating role of cortisol and sAA response between baseline characteristics of acceptance coping with memory recall and spatial orientation with decision-making performance, though the relationships remain uncertain and require future research.

### Limitations

The strengths of this study included the use of a novel VR that was created to simulate high stress military environments, which are hard to replicate in lab-based studies (Lieberman et al., 2006). This study also included a comprehensive battery of baseline assessments based on previous research to expand on current gaps in the literature. Biological specimen collection happened at five timepoints over the course of the study to examine the physiological response to the scenario, providing more insight into the stress elicited from this scenario and subsequent recovery in an active-duty population. However, a number of limitations must carefully be considered when interpreting the results of this analysis. First, the sample size of this analysis was small and the population relatively homogeneous. Therefore, generalization of results outside of a male, active-duty Soldier population should be limited, and given known differences in stress reactivity and cognitive functioning (Kudielka & Kirschbaum, 2005; Weiss et al., 2003), future research should expand to larger sample sizes and more diverse populations of military personnel. Similarly, relationships found in this study are purely correlational in nature, and therefore more research is needed before creating actionable steps toward things like targeted interventions. Third, biological sample collections were not controlled for based on waking times and therefore effects of sleep–wake cycles cannot be accounted for. Cortisol decreased following the scenario and continued to decrease throughout the remainder of the study, likely due to the diurnal pattern of salivary cortisol (Hucklebridge et al., 2005). Fourth, the overall number of variables assessed in this study exceeded the number of observations, increasing dimensionality and the risk of Type I errors. To reduce this risk, highly correlated variables were removed, and separate models were run for each domain using FDR corrections. Finally, participants did not complete a familiarization session of the cognitive or psychomotor assessments prior to baseline testing due to schedule constraints. Previous literature report using familiarization periods for a few of the tasks completed in this analysis (Beckner et al., 2021; Connaboy et al., 2019). Without familiarization, participants may have misunderstood the test processes or exhibited performance variability that did not accurately reflect their cognitive abilities with sufficient practice.

### Conclusions

These results demonstrated that psychological variables, mostly coping skills, are the most frequently associated with performance across three military-relevant VR tasks. There is also preliminary evidence that physiological biomarkers of HPA axis and SAM activity may mediate the relationship between coping skills and military-style task performance as well as cognitive function and task performance. These relationships should be explored more and possibly expanded on to include other biomarkers that are known to be associated with stress, cognition, and performance. The military continues to strive for the best and newest methodologies for training military personnel in realistic scenarios that mimic the stress and uncertainty that will be encountered in field environments, which the DeMUS task has been validated to do (Brunyé & Giles, 2023). The present study expanded on previous DeMUS research to examine what baseline psychological, cognitive, and physical variables are associated with performance on the DeMUS scenario, and these results provide preliminary evidence to support future research into the relationships between coping skills and military task performance.

## Data Availability

The datasets analyzed during the current study are available from the corresponding author on reasonable request.
